# Utility of multivessel Doppler assessment for predicting adverse neonatal outcome in late-onset intrauterine growth restriction

**DOI:** 10.1590/1806-9282.20250309

**Published:** 2026-05-01

**Authors:** Pakize Özge Karkin, İbrahim Halil Kalelioğlu

**Affiliations:** 1 University of Health Sciences, Istanbul Kanuni Sultan Süleyman Training and Research Hospital, Department of Obstetrics and Gynecology – Istanbul, Turkey.; 2 Istanbul University, Istanbul Faculty of Medicine – Istanbul, Turkey.

**Keywords:** Fetal growth retardation, Doppler sonography, Uterine artery, Umbilical artery, Middle cerebral artery, Placental circulation

## Abstract

**OBJECTIVE:**

The aim of this study was to investigate the change of Doppler measurements among late-onset intrauterine growth restriction and appropriate for gestational age fetuses and to estimate the neonatal adverse outcome in late-onset intrauterine growth restriction during longitudinal Doppler assessments.

**METHODS:**

This study was conducted at a tertiary referral hospital with 50 appropriate for gestational age and 50 late-onset intrauterine growth restriction fetuses. Late-onset intrauterine growth restriction was defined as the detection and diagnosis of growth restriction from the 32nd week of gestation. Doppler assessments were performed longitudinally for the uterine arteries, umbilical artery, and middle cerebral artery from 32 weeks of gestation until delivery. The cerebroplacental ratio was calculated for each analysis. Neonatal outcomes were recorded after delivery. Statistical analysis was performed, and a p<0.05 was considered statistically significant.

**RESULTS:**

Compared with appropriate for gestational age, umbilical artery pulsatility index and adverse neonatal outcomes were higher in late-onset intrauterine growth restriction (p<0.001, p<0.001), while term middle cerebral artery pulsatility index and term cerebroplacental ratio were lower (p=0.013, p<0.001). According to adverse neonatal outcomes in late-onset intrauterine growth restriction, term uterine arteries pulsatility index and pre-term umbilical artery pulsatility index were higher (p=0.002, p=0.013); middle cerebral artery pulsatility index and term cerebroplacental ratio were lower but without statistical significance.

**CONCLUSION:**

Umbilical artery pulsatility index, term middle cerebral artery pulsatility index, and cerebroplacental ratio are significantly different between appropriate for gestational age and late-onset intrauterine growth restriction fetuses; however, only increased uterine arteries pulsatility index on term Doppler scans may be useful in detecting adverse neonatal outcomes in late-onset intrauterine growth restriction.

## INTRODUCTION

Intrauterine growth restriction (IUGR) refers to a condition in which estimated fetal weight or abdominal circumference is under the tenth percentile by sonography^1^. This progressive disorder, which can lead to adverse outcomes such as perinatal morbidity, stillbirth, neonatal mortality, and postnatal neurodevelopmental problems, is seen at a prevalence of up to 10% in the population. IUGR is more common as a late-onset form with a prevalence of 5–10%^2^. The main problem in late-onset IUGR (L-IUGR) is patient management. Although Doppler evaluations of specific vessels are routinely performed for early-onset IUGR, there is no universally approved consensus for Doppler assessments of L-IUGR.

Fetal multivessel hemodynamic abnormalities may be associated with L-IUGR. Since there is no consensus on management, prolonging gestation time with serial multivessel Doppler assessments appears to be the only safe approach.

This study had two aims. The first aim was to examine changes in Doppler measurements for the uterine, umbilical, middle cerebral arteries, and cerebroplacental ratio in L-IUGR and appropriate for gestational age (AGA) fetuses during longitudinal assessments. The second aim was to investigate whether these Doppler measurements could be used to predict adverse neonatal outcomes in IUGR fetuses.

## METHODS

This prospective longitudinal study was conducted at Istanbul University, Istanbul Faculty of Medicine, a tertiary referral hospital. This hospital’s patient population consists primarily of high-risk pregnancies, multiple pregnancies, and fetal congenital malformations. Low-risk pregnancies are rarely followed at this tertiary referral hospital.

Between December 2015 and May 2016, patients were enrolled in research after approval from the ethics committee of Istanbul University, Istanbul Faculty of Medicine (number 2015/1992). To be eligible for the study, patients had to have a singleton pregnancy, have normal fetal anatomy, not have delivered before 34 weeks, and have been evaluated with at least two serial longitudinal Doppler evaluations. Eligible patients were divided into two groups as IUGR and AGA. Intrauterine growth restriction was defined as estimated fetal weight (EFW) below the 10th percentile and/or abdominal circumference below the 3rd percentile. The Hadlock-4 formula was used to calculate EFW^3^.

Among the fetuses diagnosed with IUGR, only late-on-set fetuses diagnosed at the 32nd week of gestation and later were selected^4,5^.

To be included in the AGA group, sonographic measurements had to be appropriate for their gestational age, and the estimated fetal weight centile had to be between the 10th and 90th percentiles. Pregnancies of 32 weeks and above were enrolled to study. Gestational age was confirmed with first-trimester crown-rump length.

Exclusion criteria for both groups were: (a) fetal congenital malformation, (b) intrauterine infection in the current pregnancy, (c) multiple gestation, (d) preeclamptic patients, and (e) chronic or gestational hypertensive diseases.

Of the 127 singleton pregnancies examined for the study, 58 met the entry criteria for the IUGR group. As the study continued among 58 IUGR pregnancies, 1 chose to change hospital and opted out of the study, 6 developed preeclampsia, and 1 delivered before 34 weeks of gestation.

Of the 69 AGA pregnancies, 9 withdrew from the study, 3 developed gestational hypertension near term, 2 delivered before the 34th week of gestation, and 5 could not undergo serial Doppler evaluation.

**Figure 1:**
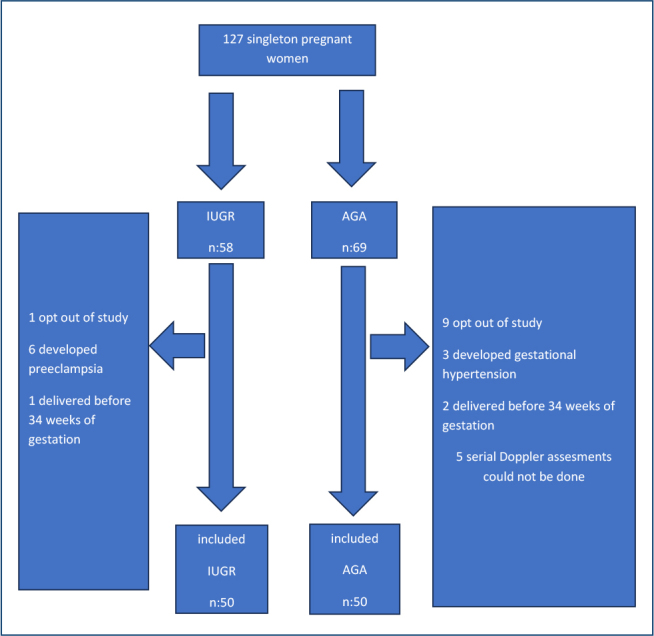
The flowchart of the study population.

Recruitments were closed for the research when the IUGR and AGA groups reached the number of 50, separately (Figure 1).

Biometry, amniotic fluid index, and placental region assessment were performed in both groups, and at least two serial Doppler examinations were performed until delivery for the whole study population.

### Doppler examinations

All ultrasound scans were performed by the same operator with the Voluson® 730 Expert (GE Healthcare, Wauwatosa, WI, USA) ultrasound imaging device. Images and Doppler recordings were obtained while the fetus was neither breathing nor moving.

All pulsed-wave Doppler measurements were recorded after three or more consecutive uniform waveforms, with an insonation angle close to zero degrees and always below 30 degrees. Doppler analysis of uterine arteries (Ut), umbilical artery (UA), and middle cerebral artery (MCA) was performed, and cerebroplacental ratio (CPR) was calculated in every single visit.

A Doppler study of the uterine artery was performed after placing the ultrasound probe on the lower quadrant of the abdomen, where the ascending branch just crosses the external iliac artery. The pulsatility index (PI) of the uterine artery was obtained. After examining the uterine arteries for both sides, the mean value of the uterine arteries was calculated by adding the values of both sides and dividing by two.

UA PI was recorded from the free-floating part of the umbilical cord.

MCA measurements were obtained at the level of the circle of Willis, in the transverse view of the fetal head, from the transducer site.

CPR was calculated by dividing MCA PI by UA PI.

Doppler examinations were performed weekly until the 37th week of gestation. After reaching term, a Doppler assessment was done twice-weekly. All included women had Doppler measurements at least 1 week before delivery.

### Delivery

Timing and type of delivery were managed by chief clinicians who were following up pregnancies. Indications for delivery were a biophysical profile (BPP) score ≤4, a BPP score persisting at 6 within 12 h, and fetal distress. Otherwise, in this clinic, labors are induced at the 38th week of gestation for IUGR and at 40+6 weeks of gestation for AGA.

### Neonatal outcomes

Neonatal outcomes such as Apgar scores, birth weight and height, and umbilical cord blood values were recorded.

Adverse neonatal outcome was defined as the occurrence of at least one of the following: low Apgar score, abnormal umbilical cord pH, abnormal base excess, tachypnea of the newborn, respiratory distress syndrome, necrotizing enterocolitis, sepsis, and neonatal intensive care unit (NICU) admission.

We defined low Apgar score as fifth-minute Apgar score <7, abnormal pH as umbilical cord arterial pH<7.1, and abnormal base excess (BE) as BE<(-12).

### Statistical analysis

Statistical analysis was performed using Statistical Package for Social Sciences (IBM SPSS Statistics for Windows, Version 21.0. Armonk, NY: IBM Corp.).

Relationships between categorical variables were evaluated with Pearson’s chi-squared or Fisher’s exact test. Shapiro-Wilk test was used for the distribution of numerical variables. As the mean and standard deviation were given for normal distribution, for abnormal distribution of numerical variables, median, minimum and maximum values were given. To compare mean values of numerical variables for two categories, Student’s t-test and to compare median values Mann-Whitney U test were used, respectively. A probability value of p<0.05 was deemed significant.

## RESULTS

Of the 127 pregnant women examined, after the inclusion and exclusion criteria, 50 were included in the L-IUGR group and 50 in the AGA group.

Maternal characteristics and perinatal outcomes are given in Table 1. The number of Doppler examinations performed on these fetuses varies from two to four.

**Table 1:** Maternal characteristics and perinatal outcomes.

Characteristics	AGA n=50	L-IUGR n=50	P
Maternal age (years)	28.5 (22–41)	29 (19–37)	0.163
Parity	1 (0–3)	1 (0–3)	0.426
Maternal BMI	28,916 (22,656–39,411)	26,773 (18,733–42,661)	0.001
Smoker	3 (6)	10 (20)	0.037
GA at first Doppler scan (weeks)	35.06±0.843	35.38±0.805	0.40
GA at last Doppler scan (weeks)	37.2±0.782	37.2±0.495	0.465
GA at delivery (weeks)	38.52±1.216	37.66±0.557	<0.001
Oligohydramnios at the last Doppler scan	0 (0)	6 (12)	0.027
Cesarean section	37 (74)	32 (64)	0.280
Birth weight (g)	3,290 (2,420–4,460)	2,367.5 (1,550–2,700)	<0.001
Apgar score at 1 min	9 (7–9)	9 (7–9)	0.173
Apgar score at 5 min	9 (8–10)	10 (7–10)	0.045
Umbilical artery pH at birth	7,318 (7,155–7,450)	7,330 (7,070–7,549)	0.649
Base excess	-1.45 (-6.2 to 1.8)	-2 (-14.1 to 1)	0.125
Female fetus	26 (52)	23 (46)	0.548
Adverse neonatal outcome	2 (4)	21 (42)	<0.001

Data are given as mean±standard deviation, median (range) and n (%): number (ratio). GA: gestational age; BMI: body mass index; AGA: appropriate for gestational age; L-IUGR: late-onset intrauterine growth restriction.

While only one of the criteria specified in the method was sufficient for adverse neonatal outcome, it was found that all fetuses with adverse neonatal outcome were admitted to the NICU during postnatal follow-up.

Compared with the AGA group, IUGR fetuses were born at earlier gestational age, tended to have oligohydramnios, had lower birth weight, and had adverse neonatal outcomes. In terms of maternal characteristics, the IUGR group had lower maternal body mass index and had more smoker mothers than the AGA group. The type of delivery was not statistically significant (p=0.280) (Table 1).

Doppler findings were given in Table 2. Uterine artery screening was not valuable for detecting IUGR but appeared important for detecting adverse neonatal outcomes in L-IUGR fetuses at the final screening before delivery.

**Table 2:** Longitudinal Doppler assessments of multivessels among the study population and comparison according to adverse neonatal outcome in L-IUGR.

	AGA n=50	L-IUGR n=50	p	L-IUGR n=50		p
				Adverse neonatal outcome		
				Positive n=21	Negative n=29	
Ut PI F	0.865 (0.55–2.12)	0.945 (0.6–2.48)	0.163	1.005 (0.685–1.735)	0.870 (0.6–2.48)	0.060
Ut PI L	0.845 (0.56–2.1)	0.828 (0.555–2.755)	0.831	1.120 (0.6–1.39)	0.725 (0.555–2.755)	0.002
UA PI F	0.790 (0.63–1.34)	1.015 (0.76–1.75)	<0.001	1.190 (0.79–1.75)	0.940 (0.76–1.50)	0.013
UA PI L	0.855 (0.59–1.26)	1.020 (0.75–1.51)	<0.001	1.140 (0.76–1.51)	0.930 (0.75–1.51)	0.078
MCA PI F	1.770 (0.80–2.95)	1.660 (1.05–2.73)	0.514	1.420 (1.20–2.59)	1.830 (1.05–2.73)	0.050
MCA PI L	1.665 (1.1–2.54)	1.440 (0.76–3.27)	0.013	1.390 (0.76–2.79)	1.440 (1.01–3.27)	0.262
CPR L	2.035 (0.873–3.429)	1.453 (0.559–3.554)	<0.001	1.384 (0.559–3.132)	1.526 (0.669–3.554)	0.072

Data are given as median (range). F: at first Doppler scan; L: at last Doppler scan; AGA: appropriate for gestational age; L-IUGR: late-onset intrauterine growth restriction; PI: pulsatility index; Ut: uterine artery; UA: umbilical artery; MCA: middle cerebral artery; CPR L: cerebroplacental ratio at last Doppler scan.

When we compared UA PI between AGA and L-IUGR, it was statistically significant at both scans, but when comparing adverse neonatal outcomes in the L-IUGR group, UA PI showed significance only at the first scan.

MCA PI was significantly lower in growth-restricted fetuses than in appropriate for gestational age fetuses at the last Doppler scan, but was not very useful in detecting adverse outcomes (Table 2).

Term cerebroplacental ratios for the entire study population and the L-IUGR group were given in Table 2. Doppler scanning at term for CPR was statistically significant between AGA and IUGR. CPR showed no significance in detecting adverse neonatal outcomes in L-IUGR fetuses.

## DISCUSSION

Fetal growth restriction affects approximately 3–10% of pregnancies, depending on the diagnostic threshold values used^6^. IUGR is associated with increased perinatal morbidity and mortality, and the biggest danger for fetal growth restriction is stillbirth during pregnancy follow-up. Since there is no effective in utero treatment, the cornerstone of management is to determine the optimal timing of delivery. But there is a dilemma, and this remains challenging; early and unnecessary obstetrical intervention may lead to iatrogenic preterm birth and its complications, whereas delaying delivery increases the risk of stillbirth.

Most international guidelines recommend delivery at around 37 weeks of gestation for suspected IUGR; however, this approach may expose constitutionally small but healthy fetuses to the risk of late-preterm birth^1,4,7^.

Since differential management is very important, in this study, we aimed to determine whether Doppler parameters are effective to predict adverse neonatal outcomes in L-IUGR while making a delivery time decision. Accurate Doppler assessment is essential for guiding delivery timing in L-IUGR. In our study, deterioration in Ut PI close to term was associated with adverse neonatal outcomes. This suggests that a rising Ut PI at term may help clinicians identify fetuses at higher risk and may be a useful additional criterion when deciding whether to expedite delivery. In contrast, although UA PI, MCA PI, and CPR differed significantly between AGA and L-IUGR fetuses, these parameters did not reliably predict adverse neonatal outcomes. Therefore, routine reliance on UA PI or CPR alone to trigger delivery in late-onset IUGR may be insufficient. Thus, integrating serial Ut PI measurements into routine surveillance may improve individualized decision-making for delivery in L-IUGR.

Our findings regarding Ut PI are consistent with those of Caradeux et al., who reported higher mean Ut PI at the last Doppler assessment in L-IUGR with the occurence of adverse perinatal outcomes^8^. On the other hand, Simeone et al. could not associate adverse outcomes with Ut PI increases in their study, as they defined emergency cesarean as an adverse out-come^9^. Some other studies have also reported increased Ut PI in relation to emergency cesarean section and adverse perinatal outcome^10,11^, while a recent study linked abnormal Ut PI to abnormal brain development in L-IUGR^12^. There are also conflicting results in the literature, indicating that longitudinal results of Ut PI show a slight but negligible increasing trend^13^.

The expected difference between the AGA and IUGR groups was obtained for UA PI, yet UA PI assessment was not deemed successful in predicting adverse neonatal outcomes for IUGR fetuses. The limited predictive value of UA PI in our study aligns with an expert review concluding that UA velocimetry is an unreliable predictor of adverse neonatal outcomes in L-IUGR^14^. Contrary to our findings, in a study where emergency cesarean section was considered as an adverse outcome, a possible association between increased UA PI and poor outcome was stated. Therewithal, in the same study, no relationship was found with NICU admission and UA PI^9^.

Decreased MCA PI in IUGR fetuses was an expected result due to the brain-sparing effect^15,16^. Although MCA PI showed significant differences between AGA and L-IUGR on term Doppler scans in our data, it was not useful in predicting adverse perinatal outcomes. In another study, MCA PI abnormality was associated with poor outcomes but the definition of poor outcome was related to the mode of delivery, but confusingly, in the same study, MCA PI was deemed pointless according to NICU admission^9^. Although some studies have highlighted cerebral blood-flow redistribution as a predictor of adverse outcome^17^, differences in study design and outcome definitions complicate direct comparison.

Similarly, although CPR differed significantly between AGA and IUGR fetuses in our study, it did not identify L-IUGR fetuses at risk for adverse neonatal outcomes. Likewise, according to NICU admission, CPR was not significant in another study^9^. Additionally, a meta-analysis showed higher predictive accuracy of CPR in early-onset IUGR, but showed no effectiveness for composite adverse perinatal outcomes in L-IUGR^18^.

Some studies that are conducted with term pregnancies with an unselected population also state low diagnostic accuracy for CPR in predicting adverse neonatal outcomes^19,20^. This possibly can be thought of as CPR having low sensitivity in late gestation. However, on the contrary, some studies have pointed out that CPR is a predictive ratio for adverse perinatal outcome^10,21^.

This study may have some limitations. Our study was conducted in a single tertiary referral center, which primarily manages high-risk pregnancies. As a result, the number of eligible participants was limited, which may reduce the generalizability of our findings. Nevertheless, the prospective longitudinal design and the well-defined, topic-specific patient population strengthen the reliability of our results.

## CONCLUSION

Significant Doppler differences exist between AGA and late-on-set IUGR fetuses; however, the ability of Doppler parameters to predict adverse neonatal outcomes remains inconsistent, likely due to variations in study design and definitions of adverse outcomes. In our study, UA PI, near-term MCA PI, and CPR were significantly different between groups, but only increased Ut PI near term was associated with adverse neonatal outcomes. These findings suggest that serial Ut PI Doppler assessment may help guide delivery timing in L-IUGR. Further prospective studies with larger populations are needed to confirm the clinical utility of Doppler measurements for delivery decisions in L-IUGR.

## Data Availability

The datasets generated and/or analyzed during the current study are available from the corresponding author upon reasonable request.
